# Anakinra in Pyogenic Arthritis, Acne, Pyoderma Gangrenosum, and Suppurative Hidradenitis (PAPASH) Spectrum Disorder: A Case Report and Literature Review

**DOI:** 10.7759/cureus.62247

**Published:** 2024-06-12

**Authors:** Anam Ahmad, Terry Moore

**Affiliations:** 1 Internal Medicine, St. Luke's Hospital, Chesterfield, USA; 2 Internal Medicine, Saint Louis University School of Medicine, St. Louis, USA

**Keywords:** pyoderma gangrenosum, hidradenitis suppurativa, anakinra, autoinflammatory, autoinflammatory disease, ptspip 1, papash syndrome

## Abstract

Pyogenic arthritis, acne, pyoderma gangrenosum, and suppurative hidradenitis (PAPASH); pyogenic arthritis, pyoderma gangrenosum (PG), and acne; PG, acne, hidradenitis suppurativa; and PG, acne, spondylarthritis (PASS) are all part of a spectrum of autoinflammatory disorders that share similar pathogenesis. They are related to various mutations in the proline-serine-threonine phosphatase interacting protein 1, leading to dysregulation of the innate immune system and overproduction of interleukin (IL)-1, IL-17, and IL-23 and tumor necrosis factor (TNF)-α. Targeting these cytokines with biologics plays an important role in treatment. Here, we are describing the case of a young male with PAPASH syndrome who was treated with TNF-α and IL-1 inhibitor.

## Introduction

Pyogenic arthritis, acne, pyoderma gangrenosum, and suppurative hidradenitis (PAPASH) is a tetrad of pyogenic arthritis pyoderma gangrenosum (PG), acne, and suppurativa hidradenitis (HS). It is a rare autoinflammatory disorder first described in 2013; less than 15 cases have been described so far [1]. Skin manifestations can occur many years before the development of arthritis. Due to the rarity of the disease, it has diagnostic and therapeutic challenges.

The case report aims to increase awareness about PAPASH syndrome. It emphasizes that patients with HS and PG should be screened for inflammatory arthritis on every visit, as our patient had HS and acne more than 10 years before developing arthritis.

## Case presentation

**Figure 1:**
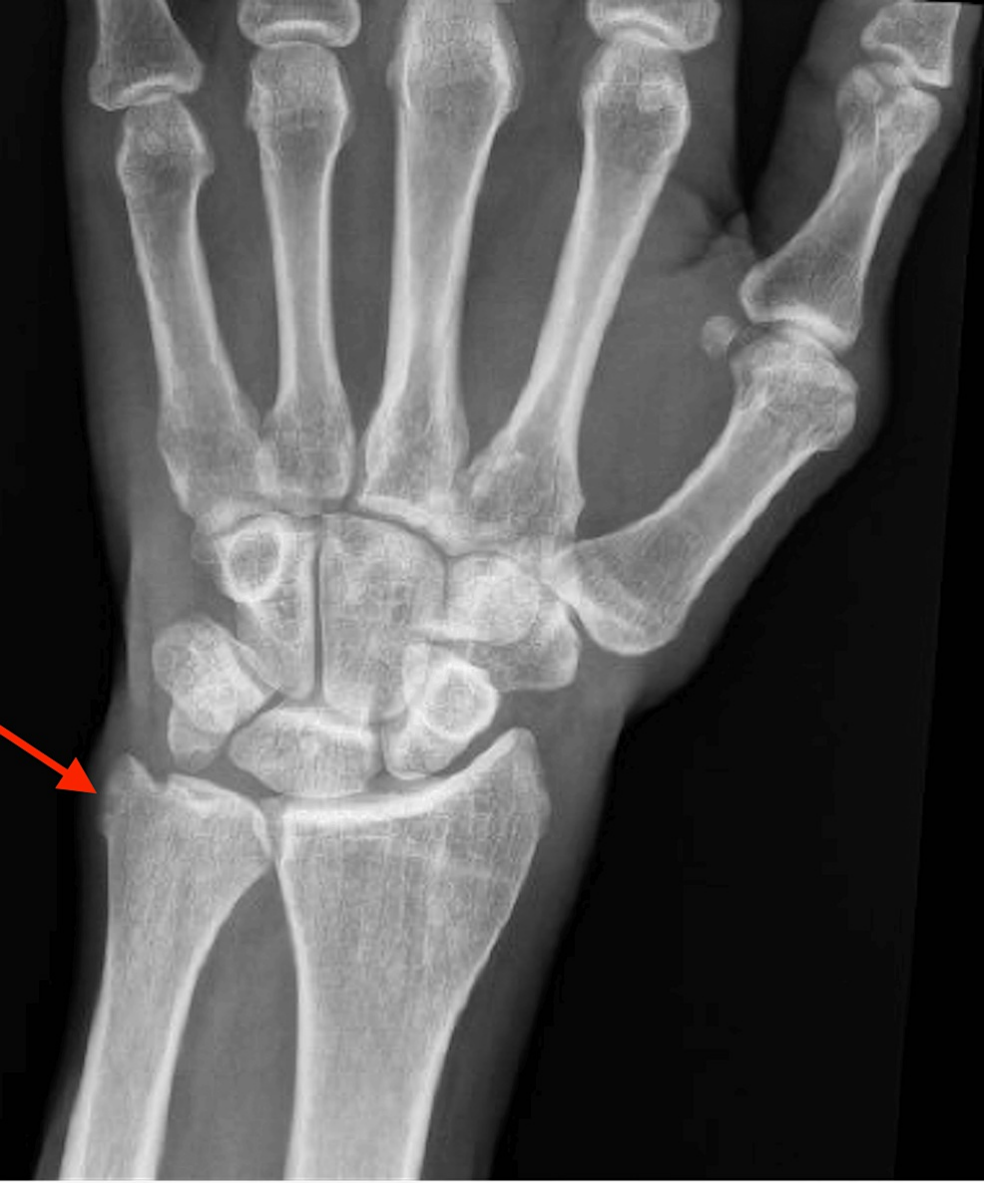
X-ray of the right hand. Red arrow reveals erosion on the ulnar styloid process, significant for inflammatory arthritis

**Figure 2:**
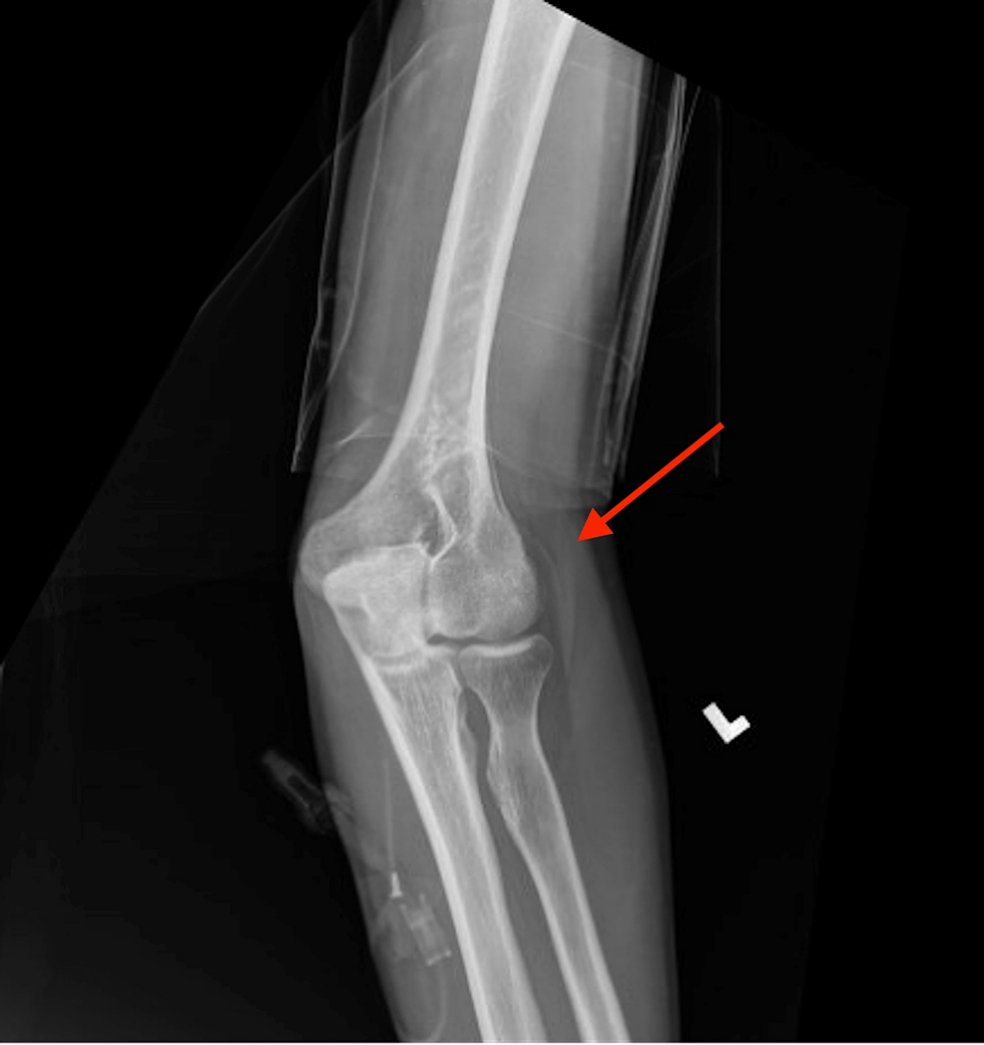
X-ray of the left elbow revealing joint effusion (red arrow)

A 35-year-old male with a past medical history, including Hurley stage 3 HS since his 20s, inflammatory acne, cribriform PG, anemia, and chronic smoking, was admitted to the hospital due to worsening polyarthralgia. He mentioned he has been experiencing progressively debilitating joint pain in multiple joints. He was experiencing episodic arthralgias in his shoulders, knees, hands, and elbows to the point that he was not able to feed himself. Pain was associated with profound stiffness all day long, confining him to bed. He had diffuse tenderness of the wrists, elbows, shoulders, and knees with a decrease in the range of motion of his elbows and knees without overt synovitis. He had healed ulcers with hypopigmentation over the legs with widespread erythematous tender nodules favoring intertriginous areas. His vitals were stable, and labs were significant for an elevated C-reactive protein of 9.1 mg/dL and an erythrocyte sedimentation rate >130 millimeters/hour with negative human leukocyte antigen B27 (HLA-B27) and rheumatoid factor but low positive anticitrullinated peptide 25 units. We ordered X-rays of the joints, which revealed changes in inflammatory arthritis, including cortical erosion of the right ulnar styloid process (Figure 1) and left elbow effusion (Figure 2). As per the constellation of symptoms of HS, PG, acne, and now inflammatory arthritis, he was diagnosed with PAPASH. He had failed infliximab (used for more than two years) and adalimumab (used for more than three years) with no improvement in his skin condition. He is currently on colchicine for his HS. After failing to respond to two tumor necrosis factor (TNF) inhibitors, we switched him to an Anakinra-interleukin (IL)-1 receptor antagonist. He initially received 100 mg subcutaneously (SC) daily, which was later increased to 200 mg (SC) daily due to a suboptimal response. This treatment was accompanied by methotrexate (MTX) 15 mg orally weekly. These changes led to an improvement in his arthralgia, joint stiffness, and joint swelling within a couple of months. His symptoms have been well controlled on the doses mentioned earlier of Anakinra and MTX for over six months now.

## Discussion

HS is an inflammatory disorder of apocrine-bearing skin characterized by recurrent, painful nodules, abscesses, formation of sinus tracts, and hypertrophic scarring. It is more prevalent in African Americans, affects females more than men, and is strongly associated with smoking and obesity [2]. PG is a neutrophilic dermatosis that presents rapidly developing, painful skin ulcers hallmarked by undermined borders and peripheral erythema [3]. Both of these skin conditions have been associated with various autoinflammatory syndromes causing recurrent sterile inflammation of skin and joints.

Various entities have been described in the literature, depending on the combination of HS, PG, and arthritis, including pyogenic arthritis, pyoderma gangrenosum, and acne (PAPA); pyoderma gangrenosum, acne, hidradenitis suppurativa (PASH); PAPASH; pyoderma gangrenosum, acne, spondylarthritis (PASS); psoriatic arthritis, PG, acne, and HS; PG, acne, ulcerative colitis; vasculitis, PG, acne, HS; and pustular psoriasis, arthritis, PG, synovitis, acne, and HS [4,5]. Due to similarities in the phenotype and pathogenesis, they are sometimes referred to as PAPA spectrum disorders [6].

PAPASH is the combination of the phenotypes of PAPA and PASH, with a mean age of diagnosis around 30-40 years [7]. Generally, the arthritis lags the skin manifestations and develops 2-15 years after the onset of skin lesions, as in our patient. The arthritis shares common features with spondylarthritis but is not linked to HLA-B27 [8]. It has been associated with inflammatory bowel diseases and inflammatory eye diseases.

PAPASH, along with other PAPA spectrum disorders, shares a common pathogenesis. A wide range of mutations of the PSTPIP1 gene and mutations in the Mediterranean fever gene are attributed to decreased inhibition of inflammasomes [4]. Inflammasomes are cytosolic multiprotein complexes that contain the caspase 1 enzymes that cleave the proinflammatory cytokine precursors, such as IL-1, IL-18, and TNF, to active form [9]. Increased production of these cytokines upregulates the innate immune system and can cause neutrophil-rich sterile inflammation of skin and joints.

Once diagnosed, TNF inhibitors-infliximab, adalimumab, or etanercept-have been used with good response [10,11]. For severe cases of HS, IL-1 inhibitors like Anakinra and canakinumab have shown good response [12,13]. As IL-17 has also been involved in the pathogenesis of PAPASH, trials of IL-17 inhibitors have, in some cases, shown efficacy [14]. Our patient had a better response to Anakinra rather than the TNF inhibitors.

Patients with PAPASH with severe skin conditions and debilitating pain have impaired quality of life, so getting help from pain management and psychiatrists, along with working on weight loss, social support groups, and smoking cessation, helps improve quality of life [15].

## Conclusions

This case highlights the importance of recognizing symptoms, signs, and treatment strategies of inflammatory arthritis in patients with HS and PG and using Anakinra in resistant cases. Physicians should be aware of musculoskeletal manifestations in patients with acne, HS, and PG; they should evaluate these symptoms on follow-up visits, as delays in diagnosis and treatment can lead to persistent inflammation and joint destruction. This article uniquely highlights Anakinra's efficacy in cases resistant to TNF inhibitors, supported by the patient's positive response to this treatment. We suggest further research and follow-up studies to validate these findings in larger patient cohorts.
